# Supplementary material for: By-Products Valorization: Peptide Fractions from Milk Permeate Exert Antioxidant Activity in Cellular and In Vivo Models
Source: Antioxidants (Basel). 2024 Oct 10;13(10):1221. doi: 10.3390/antiox13101221 (PMC11504225; doi:10.3390/antiox13101221)
Supplement: Supplementary file 1 [file antioxidants-13-01221-s001.zip › antioxidants-3233122-supplementary.pdf]

Supplementary Materials

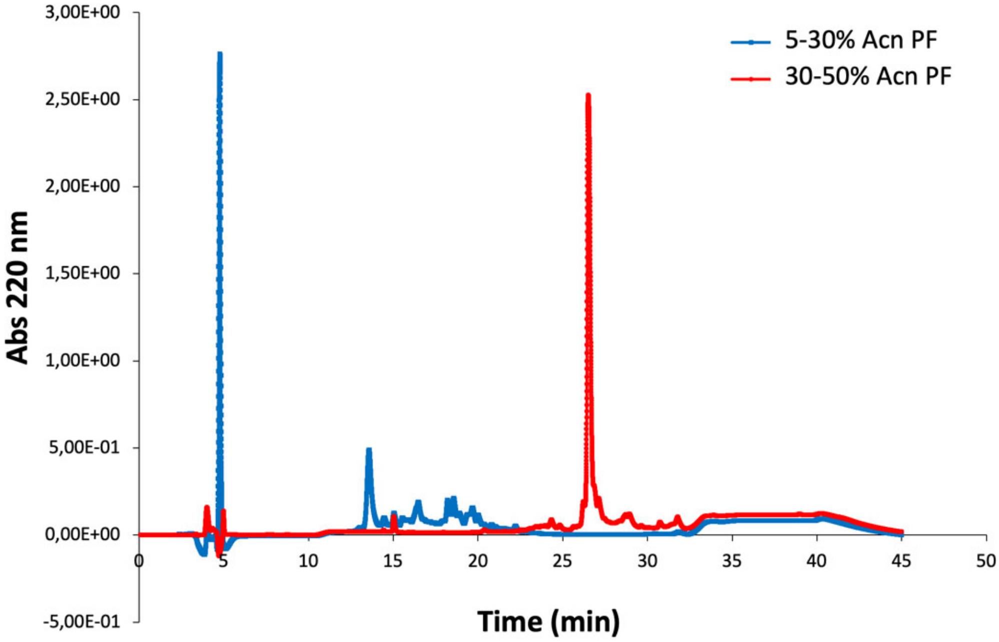

Figure S1: Chromatogram at 220 nm of the purified 5-30% and 30-50% ACN PFs with RP-HPLC.

Table S1. Peptide sequences isolated from the 5-30% ACN PF.

Sequence	Number of AA	m/z [Da]	MH+ [Da]	Derived from
**YLEQLLR**	7	467,77173	934,53618	α-S1 casein
**FFVAPFPE**	8	477,24203	953,47679	α-S1 casein
**VLNENLLR**	8	485,78772	970,56816	α-S1 casein
**LNENLLRF**	8	509,78784	1018,56841	α-S1 casein
**APFPEVFGK**	9	496,26614	991,52501	α-S1 casein
**GQVWEESLK**	9	538,27539	1075,54350	Lactoperoxidase
**TEDELQDKI**	9	545,76678	1090,52629	β casein
**LGYLEQLLR**	9	552,82422	1104,64116	α-S1 casein
**VLNENLLRF**	9	559,32220	1117,63713	α-S1 casein
**TKVIPYVRY**	9	380,22626	1138,66422	α-S2 casein
**KVIPYVRYL**	9	575,85339	1150,69951	α-S2 casein
**FVAPFPEVFG**	10	555,28723	1109,56719	α-S1 casein
**APFPEVFGKE**	10	560,78741	1120,56755	α-S1 casein
**HQGLPQEVLN**	10	567,79895	1134,59062	α-S1 casein
**YQEPVLGPVR**	10	579,32025	1157,63323	β casein
**DRITGGKDFR**	10	388,87573	1164,61264	Lipoprotein lipase
**PLSILKEKHL**	10	589,36908	1177,73088	Glycosylation-dependent cell adhesion molecule 1 (GLCM1)
**FPEVFGKEKV**	10	590,32385	1179,64043	α-S1 casein
**ILWESASLLR**	10	594,34363	1187,67998	Complement C3
**AVPYPQRDmP**	10	595,28778	1189,56828	β casein
**IVLNPWDQVK**	10	606,34290	1211,67851	α-S2 casein
**QEVLNENLLR**	10	614,33820	1227,66911	α-S1 casein
**GYLEQLLRLK**	10	411,58377	1232,73676	α-S2 casein
**LTEEEKNRLN**	10	623,32599	1245,64470	α-S2 casein
**TKLTEEEKNR**	10	416,55768	1247,65848	α-S2 casein

**TKVIPYVRYL**	10	626,37726	1251,74724	α-S2 casein
**YLGYLEQLLR**	10	634,35657	1267,70586	α-S1 casein
**YDDRNEPVLR**	10	426,21420	1276,62806	Mucin 15
**AAPAGAAIQSR**	11	506,78018	1012,55309	Polymeric immunoglobulin receptor
**YSGSSKALVST**	11	550,28632	1099,56536	Polymeric immunoglobulin receptor
**LPAPELGPRQA**	11	574,82483	1148,64238	Protein canopy homolog 3
**QPTNQVVANAK**	11	585,31738	1169,62749	Perilipin
**LSQSKVLPVPQ**	11	598,35522	1195,70317	β casein
**SSSEESITRIN**	11	611,79974	1222,59221	β casein
**LLYQEPVLGPV**	11	614,35345	1227,69963	β casein
**FVAPFPEVFGK**	11	619,33472	1237,66216	α-S1 casein
**SKVKEAmAPKH**	11	414,56058	1241,66718	β casein
**APFPEVFGKEK**	11	624,83472	1248,66216	α-S1 casein
**QPQSQNPKLPL**	11	625,34900	1249,69072	GLCM1
**LEIVPNSAEER**	11	628,82794	1256,64861	α-S1 casein
**LYQEPVLGPVR**	11	635,86176	1270,71623	β casein
**LPQEVLNENLL**	11	641,35706	1281,70683	α-S1 casein
**LPLSILKEKHL**	11	323,45923	1290,81508	GLCM1
**LPIIQKLEPQI**	11	646,40247	1291,79766	Perilipin
**VDmESTEVFTK**	11	651,30084	1301,59441	β casein
**HIQKEDVPSER**	11	669,34375	1337,68022	α-S1 casein
**TKLTEEEKNRL**	11	454,25302	1360,74451	α-S2 casein

**IQKEDVPSERY**	11	682,34705	1363,68682	α-S1 casein
**KLTEEEKNRLN**	11	687,37299	1373,73870	α-S2 casein
**QEVLNENLLRF**	11	687,87323	1374,73918	α-S1 casein
**KTKLTEEEKNR**	11	459,25595	1375,75330	α-S2 casein
**TEEEKNRLNFL**	11	696,86035	1392,71343	α-S2 casein
**YSGSSKALVSTL**	12	606,82758	1212,64787	Polymeric immunoglobulin receptor
**GVSKVKEAmAPK**	12	420,90417	1260,69797	β casein
**EPVLGPVRGPFP**	12	632,85626	1264,70525	β casein
**YQEPVLGPVRGP**	12	656,35730	1311,70732	β casein
**ALLDPSFFAKES**	12	662,84357	1324,67986	Polymeric immunoglobulin receptor
**VPPFLQPEVmGV**	12	664,85046	1328,69365	β casein
**GLPQEVLNENLL**	12	669,86737	1338,72746	α-S1 casein
**SLVYPFPGPIHN**	12	670,85394	1340,70061	β casein
**APFPEVFGKEKV**	12	449,91498	1347,73038	α-S1 casein
**VAPFPEVFGKEK**	12	674,36920	1347,73113	α-S1 casein
**LPIIQKLEPQIA**	12	681,92206	1362,83684	Perilipin
**HQGLPQEVLNEN**	12	689,34235	1377,67742	α-S1 casein
**TVDmESTEVFTK**	12	701,82416	1402,64104	α-S2 casein
**VDmESTEVFTKK**	12	477,23459	1429,68921	α-S2 casein

**ILNKPEDETHLE**	12	479,91208	1437,72168	GLCM1
**LPQEVLNENLLR**	12	719,40698	1437,80669	α-S1 casein
**NGQVWEESLKRL**	12	486,92834	1458,77048	Lactoperoxidase
**TKLTEEEKNRLN**	12	737,89697	1474,78667	α-S2 casein
**KEmPFPKYPVEP**	12	739,37433	1477,74138	β casein
**HIQKEDVPSERY**	12	500,91959	1500,74421	α-S1 casein
**AGEIQNKALLDPS**	13	678,36249	1355,71770	Polymeric immunoglobulin receptor
**EPVLGPVRGPFPI**	13	689,39789	1377,78850	β casein
**VEDHIAEGSVAVR**	13	461,24094	1381,70826	Butyrophilin subfamily 1 member A1
**PIGSENSEKTTmP**	13	703,82788	1406,64849	α-S1 casein
**SLSQSKVLPVPQK**	13	705,92010	1410,83293	β casein
**YVPLGTQYTDAPS**	13	706,34100	1411,67473	α-S1 casein
**DPARVLDLGPITR**	13	474,94067	1422,80747	Glycoprotein 2
**ALPIIQKLEPQIA**	13	717,44037	1433,87346	perilipin
**VLPVPQKAVPYPQ**	13	718,42017	1435,83306	β casein
**NAVPITPTLNREQ**	13	726,89502	1452,78276	α-S2 casein
**SSRQPQSQNPKLP**	13	489,59573	1466,77265	GLCM1
**QGLPQEVLNENLL**	13	733,89679	1466,78630	α-S1 casein
**SQNPKLPLSILKE**	13	489,62393	1466,85724	GLCM1

**HQGLPQEVL NENL**	13	745,88391	1490,76055	α-S1 casein
**GLPQEVLNEN LLR**	13	747,91913	1494,83098	α-S1 casein
**IVPNSAEERL HSm**	13	749,87067	1498,73406	α-S1 casein
**AVPYPQRDm PIQA**	13	751,37860	1501,74993	β casein
**KEPmIGVNQ ELAY**	13	754,37805	1507,74883	α-S1 casein
**KYPVEPFTES QSL**	13	762,88373	1524,76018	β casein
**KTVDmESTEV FTK**	13	510,91730	1530,73734	α-S2 casein
**FLLYQEPVLG PVR**	13	765,93817	1530,86907	β casein
**LPQEVLNENL LRF**	13	792,94165	1584,87602	α-S1 casein
**HIQKEDVPSE RYL**	13	538,61414	1613,82785	α-S1 casein
**TKLTEEEKNRL NF**	13	541,28979	1621,85483	α-S2 casein
**KEmPFPKYPV EPF**	13	812,90881	1624,81035	β casein
**EPVLGPVRGP FPII**	14	745,94128	1490,87529	β casein
**VmGVSKVKE AmAPK**	14	377,45618	1506,80288	β casein
**NPIGSENSEK TTmP**	14	760,84949	1520,69170	α-S1 casein
**LSLSQSKVLPV PQK**	14	508,64328	1523,91529	β casein
**AQPTDASAQ FIRNL**	14	766,39703	1531,78679	GLCM1
**YQEPVLGPVR GPFP**	14	778,41705	1555,82683	β casein
**ELEELNVPGEI VES**	14	778,88818	1556,76909	β casein

**SSRQPQSQNPKLPL**	14	527,29053	1579,85703	GLCM1
**VPQLEIVPNSAEER**	14	790,91870	1580,83013	α-S1 casein
**APFPEVFGKEKVNE**	14	530,94342	1590,81571	α-S1 casein
**HQGLPQEVLNENLL**	14	802,42596	1603,84465	α-S1 casein
**LIVTQTmKGLDIQK**	14	535,30847	1603,91086	β-lactoglobulin
**IGSENSEKTTmPLW**	14	804,88409	1608,76091	α-S1 casein
**QGLPQEVLNENLLR**	14	811,94788	1622,88848	α-S1 casein
**TAIRNGQVWEESLK**	14	544,29053	1630,85703	Lactoperoxidase
**FFVAPFPEVFGKEK**	14	821,43835	1641,86943	α-S1 casein
**GLPQEVLNENLLRF**	14	821,45172	1641,89617	α-S1 casein
**YQGPIVLNPWDQVK**	14	828,94171	1656,87615	α-S2 casein
**KTVDmESTEVFTKK**	14	553,61554	1658,83207	α-S2 casein
**HIQKEDVPSERYLG**	14	557,62158	1670,85019	α-S1 casein
**EGQEQEGEEmAEYR**	14	850,84021	1700,67314	Butyrophilin subfamily 1 member A1
**TKLTEEEKNRLNFL**	14	578,98438	1734,93857	α-S2 casein

Table S2. Peptide sequences isolated from the 30-50% ACN PF.

Sequence	Number of AA	m/z [Da]	MH+ [Da]	Derived from
**LPLSILKE**	8	456,79205	912,57683	GLCM1
**LGYLEQLLR**	9	552,82465	1104,64202	α-S1 casein
**LGYLEQLLRL**	10	609,36664	1217,72600	α-S1 casein
**QEVLNENLLR**	10	614,33875	1227,67021	α-S1 casein
**TKLTEEEKNR**	10	416,55792	1247,65921	α-S2 casein
**YLGYLEQLLR**	10	634,35645	1267,70561	α-S1 casein
**FVAPFPEVFGK**	11	619,33472	1237,66216	α-S1 casein
**FFVAPFPEVFG**	11	628,82184	1256,63640	α-S1 casein
**LPLSILKEKHL**	11	430,94342	1290,81571	GLCM1
**HIQKEDVPSER**	11	446,56512	1337,68082	α-S1 casein
**LGYLEQLLRLK**	11	449,27859	1345,82123	α -S1 casein
**HLPLPLLQSWm**	11	675,86639	1350,72551	β casein
**QEVLNENLLRF**	11	687,87274	1374,73821	α -S1 casein
**YLGYLEQLLRL**	11	690,89856	1380,78984	α -S1 casein
**LGPVRGPFPIIV**	12	632,89288	1264,77849	β casein
**ALLDPSFFAKES**	12	662,84357	1324,67986	Polymeric immunoglobulin receptor
**HQGLPQEVLNEN**	12	689,34210	1377,67693	α -S1 casein
**FFVAPFPEVFGK**	12	692,86969	1384,73210	α -S1 casein
**TKLTEEEKNRLN**	12	492,26678	1474,78580	α -S2 casein
**YLGYLEQLLRLK**	12	503,63339	1508,88562	α-S1 casein
**GSKASADESLALG**	13	603,30414	1205,60100	Fibroblast growth factor-binding protein 1
**VLGPVRGPFPIIV**	13	682,42743	1363,84758	β casein
**VEDHIAEGSVAVR**	13	461,24011	1381,70578	Butyrophilin subfamily 1 member A1

**VLPVPQKAVP YPQ**	13	718,41943	1435,83159	β casein
**HQGLPQEVLN ENL**	13	745,88391	1490,76055	α -S1 casein
**GLPQEVLNENL LR**	13	747,91858	1494,82988	α -S1 casein
**FFVAPFPEVFG KE**	13	757,39093	1513,77458	α -S1 casein
**LPQEVLNENLL RF**	13	792,94177	1584,87627	α -S1 casein
**PVLGPVRGPFP IIV**	14	730,95361	1460,89995	β casein
**EPVLGPVRGPF PII**	14	745,94000	1490,87273	β casein
**HQGLPQEVLN ENLL**	14	802,42615	1603,84502	α -S1 casein
**FFVAPFPEVFG KEK**	14	547,96088	1641,86808	α -S1 casein
**GLPQEVLNENL LRF**	14	821,45282	1641,89836	α -S1 casein
**KTVDmESTEVF TKK**	14	553,61523	1658,83115	α -S2 casein
**HIQKEDVPSER YLG**	14	557,62164	1670,85038	α -S1 casein
**VPSERYLGYLE QLL**	14	840,45483	1679,90239	α -S1 casein
**TKLTEEEKNRL NFL**	14	578,98450	1734,93894	α -S2 casein
**PSERYLGYLEQ LLR**	14	868,97028	1736,93328	α -S1 casein
